# The Investigational Clinical Center: a clinical-supportive and patient-centered trial unit model. Ten years of experience through normal and pandemic times of a large pediatric trial center in Italy

**DOI:** 10.1186/s13052-021-01099-0

**Published:** 2021-07-13

**Authors:** Giuseppe Pontrelli, Marco Ciabattini, Franco De Crescenzo, Isabella Biondi, Rossana Cocchiola, Giorgia Copponi, Claudia Frillici, Francesca Molinari, Francesca Rocchi, Alessandra Simonetti, Paolo Rossi, Susanna Livadiotti

**Affiliations:** 1 Academic Department of Pediatrics (DPUO), Clinical Trial Center, Bambino Gesù Children’s Hospital, IRCCS, Rome, Italy; 2 Department of Biomedicine and Prevention, University of Rome Tor Vergata, Rome, Italy; 3 Department of Psychiatry, University of Oxford, Oxford, UK; 4 INCiPiT (Italian Network for Pediatric Clinical Trials) National Hub, Bambino Gesù Children’s Hospital, IRCCS, Rome, Italy; 5 Department of Systems Medicines, University of Rome Tor Vergata, Rome, Italy

**Keywords:** Clinical trial unit, Trial implementation, Trial management, Research staff, Pediatric clinical research, Pediatrics

## Abstract

Evidence-based medicine relies on appropriately designed, conducted and reported clinical trials (CTs) to provide the best proofs of efficacy and safety for pharmacological and non-pharmacological treatments. Modern clinical research features high complexity and requires a high workload for the management of trials-related activities, often hampering physicians’ participation to clinical trials. Dealing with children in clinical research adds complexity: rare diseases, parents or legal guardian reluctance to engage and recruitment difficulties are major reasons of pediatric trials failure.

However, because in pediatrics many treatments are prescribed off-label or are lacking, well-designed clinical trials are particularly needed. Clinical Trial Units (CTUs) are indeed an important asset in the implementation of clinical trials, but their support to investigators is limited to administrative and non-clinical tasks. In this paper we present the model of the Investigational Clinical Center (ICC) of the Bambino Gesù Children’s Hospital in Rome. The ICC includes clinicians supporting the Principal Investigators for clinical management of enrolled patients in compliance of Good Clinical Practice, the legal framework of Clinical Trials. Furthermore, we present 10 years’ experience in pediatric clinical trials and how it has been affected in 2020 by the COVID-19 pandemic. The activity of the ICC has been evaluated according to specific metrics of performance. The ICC model offers a complete support, helping investigators, patients and their families to overcome majority of barriers linked to clinical research, even in time of pandemic. We propose this organization as an innovative model for total-supportive and patient-centered clinical trial implementation.

**Supplementary Information:**

The online version contains supplementary material available at 10.1186/s13052-021-01099-0.

Clinical trials (CTs), when appropriately designed, conducted and reported, provide the best evidence of efficacy and safety for pharmacological and non-pharmacological treatments, but are increasingly expensive, complex, and need highly specialized competencies.

Trials are particularly lacking in children: in this population evidence of safety and efficacy of drugs is scarce, and off-label prescription is a common practice, with potential issues for patients’ safety [1]. Trials in children aim to determine appropriate dosage for different age groups, which feature large variability in pharmacokinetics and pharmacodynamics [2]. Developing and conducting pediatric trials poses also important challenges for specific regulatory and ethical aspects, including specific risk/benefit assessment, provision of parental informed consent and age-appropriate children assent [3].

Principal Investigators (PIs) and their close collaborators dealing with pediatric trials are often chosen by Sponsors mainly for their specialized clinical competence and for their access to the population for which the clinical trial is designed.

Performing pediatric trials involves important burdens in terms of time for non-clinical and clinical activities. It requires specific knowledge and multidisciplinary competences in terms of regulatory, ethic and scientific expertise, which may represent a barrier for clinicians busy with routine clinical duties. Indeed, clinicians are often not willing to participate in clinical trials due to many related complexities and burdens. In a survey conducted among American pediatricians, the training of the site staff in clinical research procedures resulted as one of the main barriers for participating in a trial [4]. Moreover, lack of time due to daily clinical care is also regularly reported as a significant obstacle restraining physicians from participating in clinical research [5].

Clinical Trial Units (CTUs) have been established in the last decades, including study coordinators, data managers, statisticians and personnel who help clinicians in performing administrative activities. CTU assistance does surely improve trials implementation and management, but it does not address all the barriers to participation of clinicians in trials, leaving all the clinical tasks to PIs and their close collaborators [6].

Indeed, different models have been adopted for the implementation of clinical trials:

- Standard Clinical Trial Site: trials conducted in hospital wards, PI and Sub-Investigators not supported in any of the trial activities;
- Supported Clinical Trial Site: a CTU supports PI and Sub-Investigators in administrative and non-clinical activities;
- Investigational Clinical Centre: PI and Sub-Investigators are totally supported not only in administrative activities, but also in clinical duties by physicians, nurses fully dedicated to trials.

We present in Fig. 1 a scheme of the evolution of organization models of clinical research, with the pros and cons of each model.

**Fig. 1:**
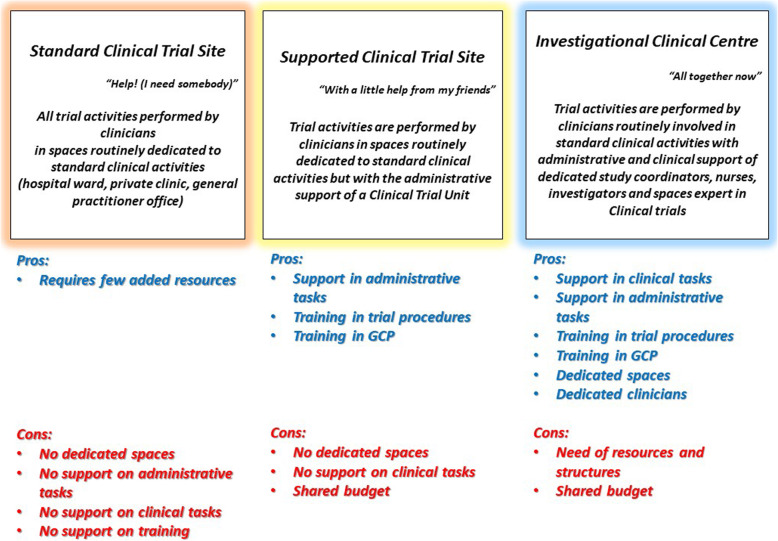
Organization models of clinical research. Pros and cons

Investigators and study nurses are key figures for all clinical trials, as they perform clinical tasks required by the protocol and assess the safety and the efficacy of the investigational treatment, the permanence of a patient in a trial, the severity and causality of adverse events (AEs). These duties, together with many other barriers associated to the growing complexity of trials procedures, and the lack of time due to standard clinical obligations, hamper the participation of clinicians to clinical trials, ultimately slowing down clinical research [5].

The “Centro Trials” of Bambino Gesù Children’s Hospital, is an archetype of Investigational Clinical Center (ICC). The ICC is led by a core team of clinicians, who support PIs and specialist sub investigators in conducting the trials and helping in the clinical management of the trial subjects. In this model, the investigators are supported by ICC’s physicians and nurses, who are confident with the clinical duties common to all trials, such as timely AEs and SAEs report with causality assessment, randomization, blinded drug administration and compliance.

Study coordinators support the start-up process, organize visits procedures, complete case report forms (CRFs) with trial patients’ data and help sponsor delegated clinical research assistants (CRA) in their monitoring visits at the IC. Furthermore, they work closely with clinical staff, providing timely information about trial’s required data.

All ICC’s personnel are trained in Good Clinical Practices (GCP) and take part in feasibility visits and site initiation visits (SIVs). They act also as trainers to PIs and their entourage for GCP and trial procedures, organize annual GCP courses for Hospital and external investigators and ultimately promote a Hospital-wide clinical research culture. Moreover, the ICC personnel offers their methodological services to various stakeholders (including independent researchers and drug companies), supporting not only the conduction but also the design and development of profit and no-profit clinical trials.

The ICC features its own dedicated spaces, providing investigators with an efficient infrastructure to recruit, perform and manage clinical trials, far from the crowd of clinical wards and ambulatories. Those spaces include dedicated rooms for medical visits, drug infusions, monitoring visits, archive, and a samples’ processing room with regularly monitored equipment (centrifuge and freezers) according to the requirements of Good Clinical Practices.

In scientific literature, there is a paucity of relevant publications regarding performance indicators of trial implementation [7]. We produced a list of metrics that could fit our purpose to properly assess the performance of organizations deputed to the implementation of clinical trials. This list also includes some metrics used in the internal ISO procedures.

We divided the metrics of performance in three main distinct areas, considering:

1. Overall activity:
  - number of new opened trials per year;
  - number of active trials per year.
2. Recruitment and retention:
  - ratio of final number of enrolled patients over expected as stated in the contract agreement per year;
  - dropout ratio (% of patients that prematurely ended their study participation after enrolment for own decision or protocol deviation) on the total of randomized patients per year.
3. Protocol Compliance:
  - percentage of randomized patients with at least one protocol violation.

The ICC of the Bambino Gesù Children’s Hospital showed a positive trend in the overall activity metrics: in the period from January 2010 and December 2020 the number of opened studies amounted to 211. The number of active studies per year from 2010 and 2020 increased almost constantly, moving from 18 active studies in 2010 to 104 active studies at the end of 2020. The number of new opened trials per year ranged from a minimum of 10 to a maximum of 31. The recruitment and retention metrics are of relevant importance, as the enrolment phase is particularly critical in pediatric trials. The average ratio of final number of enrolled patients over the expected per year was 74% (Fig. 2). The dropout ratio per year in the 2010–2020 period ranged from a minimum of 0% in 2015 to a maximum of 14.3% in 2016, for a total of 34 dropouts, mostly due of perception of lack of drug efficacy or consent withdrawal. The percentage of patients with protocol violations amounted to 0%.

**Fig. 2:**
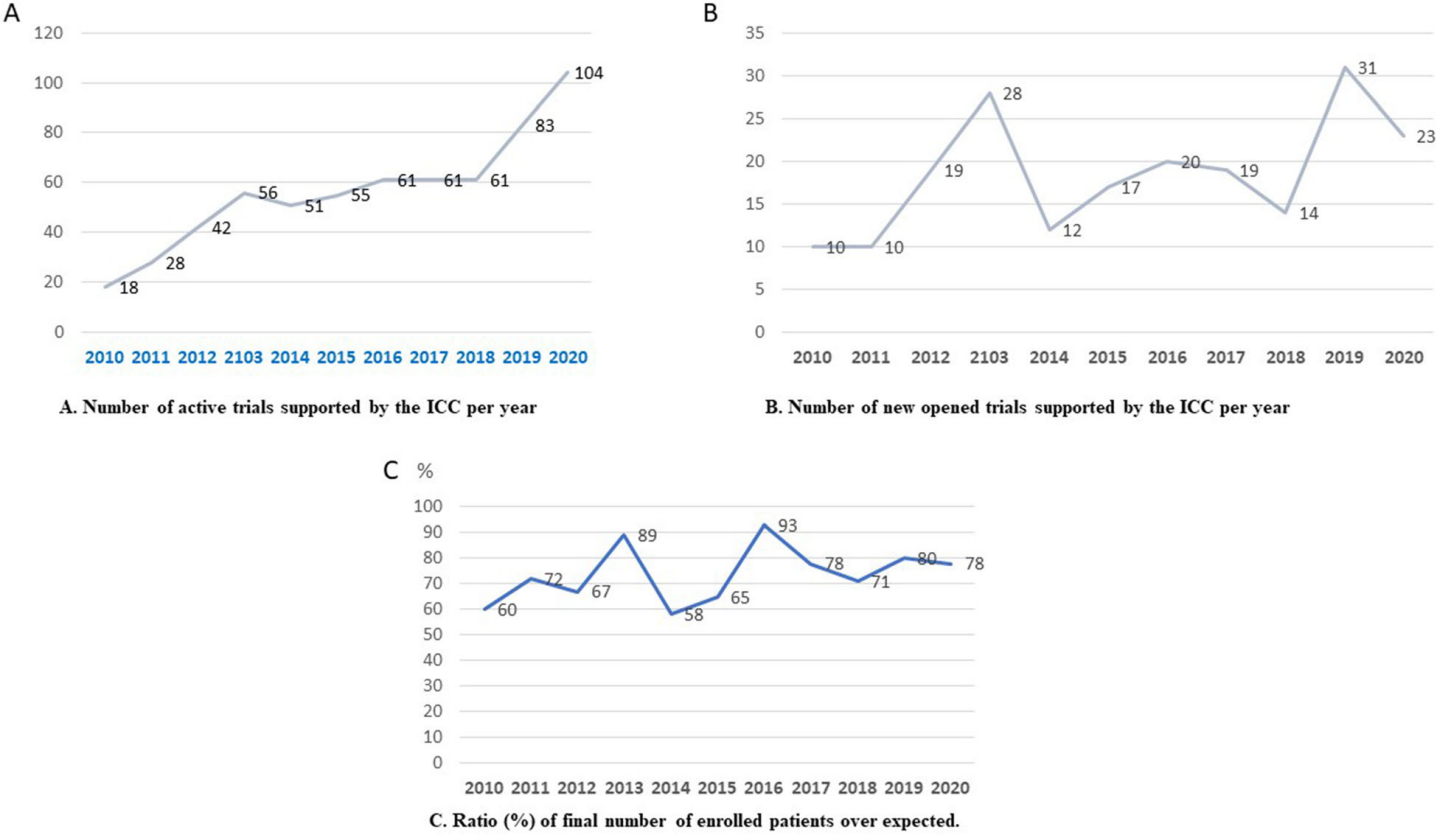
Principal indicators of ICC activity, 2010-2020

The ICC supports studies for investigational drugs in different phases of development (also Phase I), and many different therapeutic areas (see Supplementary Material S1), but not in oncology, for which there is a dedicated trial center at the Bambino Gesù Children’s Hospital.

However, it should be highlighted how not only clinicians benefit from the ICC support: first of all, it guarantees children a safe and reliable administration of experimental drugs in a caring and dedicated environment. ICC clinical personnel only deal with patients recruited in clinical trials, ensuring that a greater attention is paid to each one of them. Moreover, it provides a private place where parents and children can relate with clinicians and share their experience with other families or patients. This is important to make them feel comfortable, facilitating the building of trust between clinicians and families. Doctor-child and doctor-parent relationships are indeed essential in pediatric research, as the parents’ willingness to enroll their children in a clinical trial depends on the benefits and risks of the trial perceived during presentation of the study and informed consent and assent acquisition [8]. One of the main problems related to pediatric clinical trials’ failure is the difficulty in enrolling subjects, which often leads to issues in the trials completion [9].

During 2020, the SARS-CoV-2 pandemic determined the rise of a great number of new challenges in clinical trials implementation, related with the procedures to prevent the spread of the novel virus. Drug regulatory agencies including EMA, FDA and the Italian drug regulatory agency AIFA released guidelines for new and ongoing trials during COVID-19 public health emergency, helping CTs opening and conduction in a circumstance which gave additional burdens and hurdles for the investigators to overcome. Patients could not always come to attend the study visits at the site, each individual was scheduled at exact time in order to avoid crowd, rooms were sanitized after each visit, patients and guardians were asked to wear facial masks, safety telephone screening with body temperature assessed were performed the day before visit. According to the regulatory agencies’ recommendations, the visits at the clinical site, when possible, were replaced with phone calls and investigational drug was sent directly from the hospital pharmacy to the patient’s home. Monitoring visits were performed in remote modality, with supplemental activities of ICC study coordinators. In the case of update calls, the procedure was carried out without further authorization, while in the case of video calls that required Source Data Verification (SDV), the sponsor had to request prior authorization to the Institutional Data Protection Officer.

Despite these additional hurdles, the structure of ICC succeeded to maintain its activities, and assured continuity in clinical assistance to his patients. Only one dropout and no protocol violations occurred in 2020. Moreover, 23 new studies were started in this year.

ICC of the Bambino Gesù Children’s Hospital contributed to provide evidence for the approval of some of the most relevant drugs recently approved in the pediatric population, such for the treatment of rare diseases, including Cystic Fibrosis, Duchenne Muscular Dystrophy, Batten disease and Spinal Muscular Atrophy (Table 1).

**Table 1:**
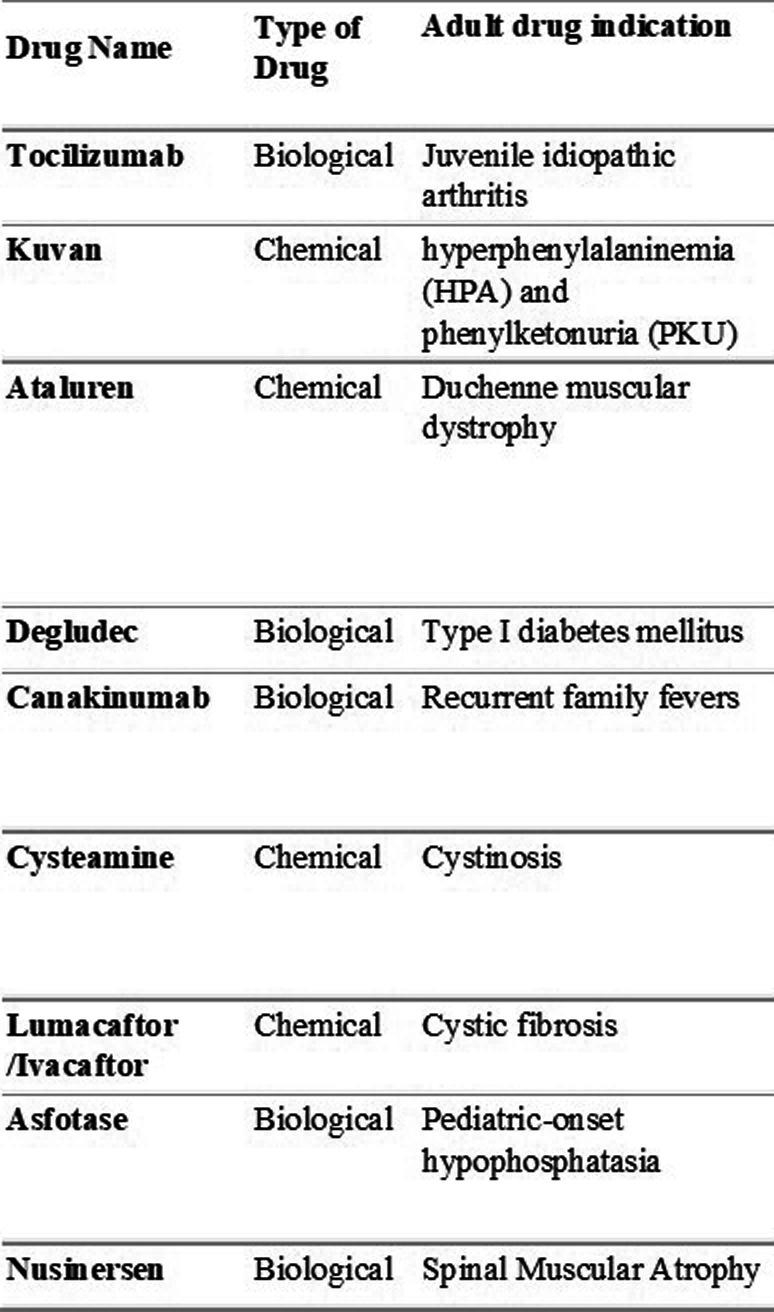
Lists of the drugs Bambino Gesù Children’s Hospital ICC contributed to approve in the pediatric population

To address the difficulties in the design and conduct of paediatric clinical trials, there is the need of trained and specialized centres, and collaborative international network, like the Collaborative Network for European Clinical Trials for Children (conect4children, c4c) in Europe, or national networks [10].. The ICC of the Bambino Gesù Children’s Hospital is one of the founding members of INCiPiT (Italian Network for Pediatric Clinical Trials), a no-profit Consortium composed by the main Italian children’s hospitals, the largest departments of pediatrics as well as national and International pediatric therapeutic networks, like c4c, coordinated by Italian institutions. The scope of INCiPiT is to foster high-quality research on drugs in children in Italy; INCiPiT aims to support the planning, conduction and completion of all types of clinical studies in the pediatric population, by providing expertise and coordinating logistical support to academic investigators as well as to pharmaceutical industries and contract research organizations.

In conclusion it has been highlighted how CTUs provide an important assistance but are not exhaustive and cannot address many barriers and issues related to clinical research [7]. The implementation of a new model in a heterogeneous international context, and with the need of a culture of innovation, specialized human resources and initial investments, can be challenging, but in our opinion, the ICC represents an improved model for clinical trials management, providing complete support to both investigators and patients, and could be a sound answer to the needs of clinical research.

## Supplementary Information

**Additional file 1: Supplementary Material S1.** Number of trials per therapeutic supported by the Bambino Gesù Children’s Hospital Investigational Clinical Center.

## Data Availability

The datasets generated and/or analysed during the current study are not publicly available due to presence of personal and confidential data.

## Abbreviations

AE: Adverse Event
CRF: Case Report Form
CTU: Clinical Trial Unit
GCP: Good Clinical Practice
ICC: Investigational Clinical Center
IMP: Investigational Medical Product
PI: Principal Investigator
SIV: Site Initiation Visit

## References

[1] Balan S, Hassali MA, Mak VS (2015). Awareness, knowledge and views of off-label prescribing in children: a systematic review. Br J Clin Pharmacol.

[2] Russo R, Capasso M, Paolucci P, Iolascon A (2011). TEDDY European Network of Excellence Pediatric pharmacogenetic and pharmacogenomic studies: the current state and future perspectives. Eur J Clin Pharmacol.

[3] Chin WW, Joos A (2016). Moving toward a paradigm shift in the regulatory requirements for pediatric medicines. Eur J Pediatr.

[4] Greenberg RG, Corneli A, Bradley J, Farley J, Jafri HS, Lin L, Nambiar S, Noel GJ, Wheeler C, Tiernan R, Smith PB, Roberts J, Benjamin DK (2017). Perceived barriers to pediatrician and family practitioner participation in pediatric clinical trials: findings from the clinical trials transformation initiative. Contemp Clin Trials Commun.

[5] Mahmud A, Zalay O, Springer A, Arts K, Eisenhauer E (2018). Barriers to participation in clinical trials: a physician survey. Curr Oncol.

[6] Gohel MS, Chetter I (2015). Are clinical trials units essential for a successful trial?. BMJ..

[7] Whitham D, Turzanski J, Bradshaw L, Clarke M, Culliford L, Duley L, Shaw L, Skea Z, Treweek SP, Walker K, Williamson PR, Montgomery AA, Site Performance Metrics for Multicenter Randomised Trials Collaboration (2018). Development of a standardised set of metrics for monitoring site performance in multicenter randomised trials: a Delphi study. Trials.

[8] Caldwell PH, Murphy SB, Butow PN, Craig JC (2004). Clinical trials in children. Lancet..

[9] Pica N, Bourgeois F. Discontinuation and nonpublication of randomized clinical trials conducted in children. Pediatrics. 2016;138. 10.1542/peds.2016-0223. 10.1542/peds.2016-0223 PMC5005019 27492817

[10] Turner MA, Hildebrand H, Fernandes RM, de Wildt S, Mahler F, Hankard R, Leary R, Bonifazi F, Nobels P, Cheng K, Attar S, Rossi P, Rocchi F, Claverol J, Nafria B, Giaquinto C (2021). The conect4children (c4c) consortium: potential for improving European clinical research into medicines for children. Pharm Med.

