# Supplementary figures and images for: The Investigational Clinical Center: a clinical-supportive and patient-centered trial unit model. Ten years of experience through normal and pandemic times of a large pediatric trial center in Italy
Source: Ital J Pediatr. 2021 Jul 13;47:156. doi: 10.1186/s13052-021-01099-0 (PMC8276228; doi:10.1186/s13052-021-01099-0)

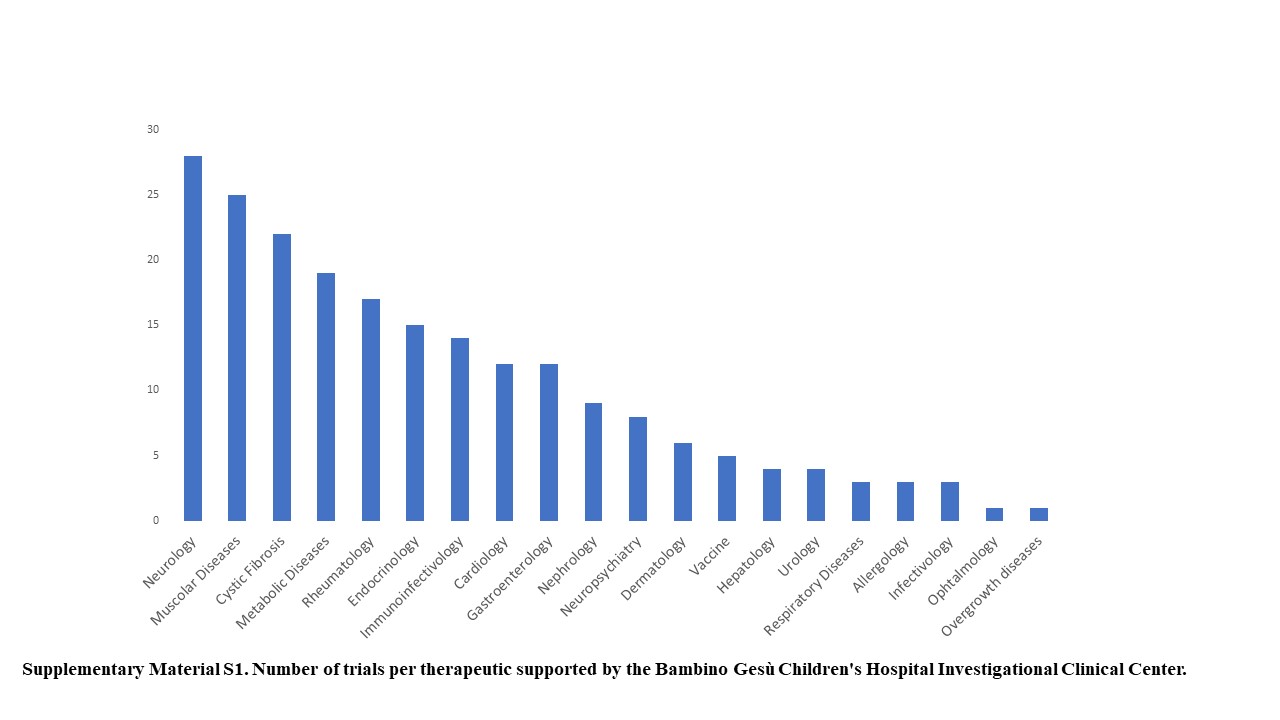

Supplement: Supplementary file 1 — Additional file 1: Supplementary Material S1. Number of trials per therapeutic supported by the Bambino Gesù Children’s Hospital Investigational Clinical Center. [file 13052_2021_1099_MOESM1_ESM.jpg]
